# Leiomyosarcoma of the inferior vena cava: a case report and review of the literature

**DOI:** 10.1186/1757-1626-3-71

**Published:** 2010-02-23

**Authors:** Venkataprasanth P Reddy, Peter J VanVeldhuizen, Gregory F Muehlebach, Reginald W Dusing, James P Birkbeck, Stephen K Williamson, Leela Krishnan, David G Meyers

**Affiliations:** 1 Departments of Hematology/Oncology, The University of Kansas Medical Center, 3901 Rainbow Blvd, Kansas City, KS, 66160, USA; 2 Cardiothoracic Surgery, The University of Kansas Medical Center, 3901 Rainbow Blvd, Kansas City, KS, 66160, USA; 3 Department of Radiology, The University of Kansas Medical Center, 3901 Rainbow Blvd, Kansas City, KS, 66160, USA; 4 Department of Cardiology, The University of Kansas Medical Center, 3901 Rainbow Blvd, Kansas City, KS, 66160, USA; 5 Department of Radiation Oncology, The University of Kansas Medical Center, 3901 Rainbow Blvd, Kansas City, KS, 66160, USA

## Abstract

A 68-year-old white female presented with two years of progressively worsening dyspnea. Echocardiography revealed a large right atrial mass and partial obstruction of the inferior vena cava. Further imaging revealed a cystic dense mass in the inferior vena cava and right atrium. Immunohistochemical stains were consistent with leiomyosarcoma. Intraoperatively, the tumor was noted to originate from the posterior aspect of the inferior vena cava. The patient underwent successful resection of the mass. Adjuvant radiation therapy was completed. The patient's dyspnea gradually improved and she continues to remain disease free five years post-resection.

## Introduction

Leiomyosarcomas of vascular origin are rare tumors arising most frequently from the inferior vena cava (IVC). Only about 200 cases worldwide have been reported since 1871 [1]. Leiomyosarcomas arising in the IVC are most frequently seen in the sixth decade, with a female predominance [2]. Symptoms are nonspecific and may precede the diagnosis by several years [3]. Diagnosis is often challenging as patients may present with non-specific complaints such as dyspnea, malaise, weight loss, abdominal pain, or back pain [3]. Computed tomography (CT), magnetic resonance imaging (MRI), individually or in combination with cavography, ultrasound (US), and echocardiography, allow an early and accurate preoperative diagnosis [3]. We describe an unusual case of a leiomyosarcoma of the IVC and review the literature.

## Case presentation

Our patient is a 68-year-old white female with a two-year history of progressive dyspnea and a persistent non-productive cough. Past medical history included hypertension, lower extremity neuropathy of unknown etiology, and interstitial cystitis. She had a remote history of tobacco use but no exposures to other toxins. Family history was positive for stroke, diabetes, coronary artery disease, and gastric cancer. Physical exam was normal except for bilateral trace lower extremity edema. No jugular venous distension, heart murmur, or tumor plop was present.

Four months previously, a CT scan of the chest, pulmonary function tests, and a pulmonary angiogram were normal. A sleep study showed no apnea but nocturnal hypoxia prompting a prescription for home O2 at night.

**Figure 1:**
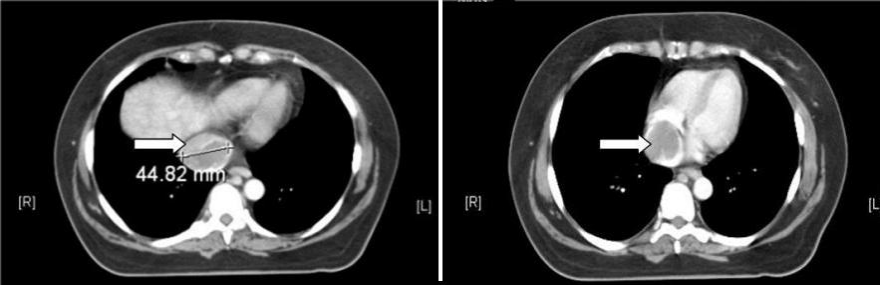
**CT Abdomen showing leiomyosarcoma in the inferior vena cava**. On the left (a), note that the transverse diameter of the mass is approximately 4.5 cm. On the right (b), the tumor mass is seen clearly within the right atrium.

At the time of re-evaluation, a transthoracic echocardiogram (TTE) demonstrated a normal ejection fraction of 60% with normal chamber dimensions but showed a large right atrial mass with high flow velocity around the echodensity. A small patent foramen ovale (PFO) was also present. Estimated pulmonary artery pressure was 44 mmHg. A transesophageal echocardiogram confirmed the large immobile right atrial mass, which involved the IVC. A CT scan revealed a cystic dense mass in the IVC and the right atrium with no evidence of pulmonary emboli or metastases (Figure 1). The abdomen and pelvis were negative with no evidence of renal cell carcinoma, which can also present with IVC involvement.

**Figure 2:**
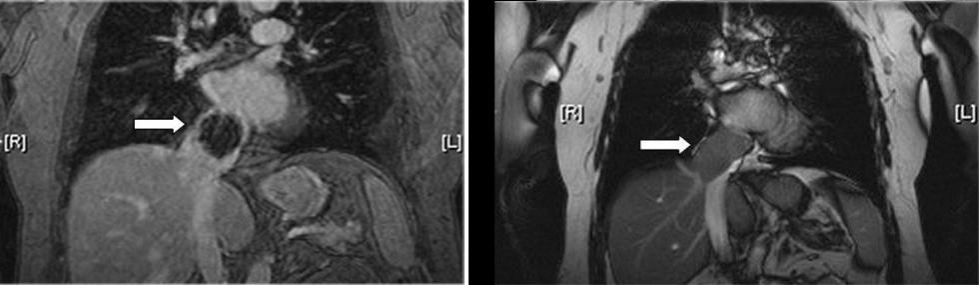
**MRA/MRI of the mass in the inferior vena cava**. On the left (a), MRA highlighting the tumor mass in the inferior vena cava. On the right (b), MRI highlighting the tumor mass in the inferior vena cava.

There was mild cerebral atrophy without evidence of intracranial masses on a CT scan of the head. A combined PET (positron emission tomography)/CT scan of the chest showed a partially contrast-enhancing intraluminal mass within the intrahepatic and suprahepatic inferior vena cava extending into the right atrium. There was no extension into the hepatic veins. The mass markedly narrowed the suprahepatic inferior vena cava and right atrium with only a small amount of residual lumen noted medially. There was diffuse heterogeneity of the liver without evidence of mass lesion, suggesting hepatic venous congestion. The PET imaging demonstrated uptake in the tumor mass consistent with malignancy but no evidence of metastatic disease. Magnetic resonance angiography (MRA/MRI) showed that the large mass centered at the confluence of the inferior vena cava and right atrium and extended into the right atrium (Figure 2). The mass extended 2.5 cm below the diaphragm. The widest transverse dimension was 4.5 cm and the greatest length obliquely was 5.6 cm. There was collateral venous flow with dilatation of the azygous vein and lumbar collaterals.

**Figure 3:**
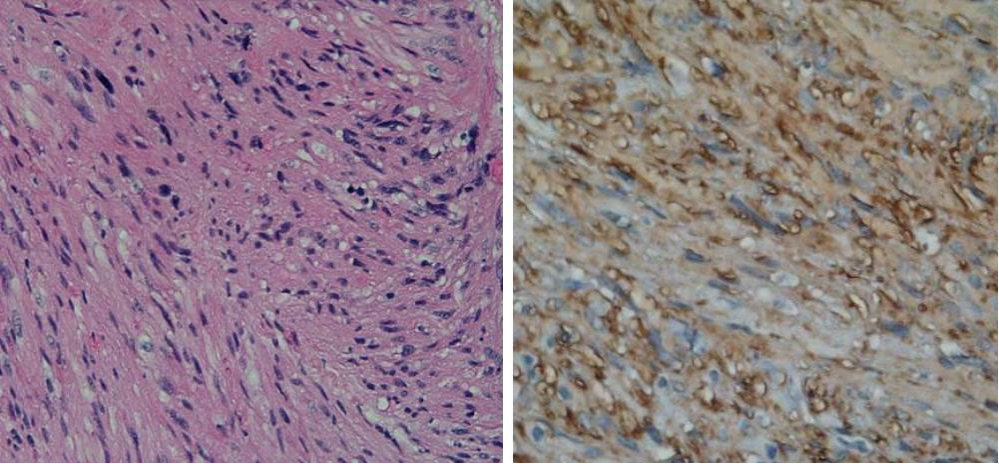
**Histology and immunohistochemistry of leiomyosarcoma of the IVC**. H&E (a, ×200) stain showing spindle cells. Tumor is positive for desmin (b, ×200, brown) indicating smooth muscle lineage consistent with leiomyosarcoma.

A fluoroscopically guided biopsy was preformed and histology revealed spindle shaped cells. Immunostains for smooth muscle actin and desmin were positive and those for melanoma and cytokeratin were negative, consistent with the diagnosis of leiomyosarcoma (Figure 3).

Intraoperatively, the tumor was noted to originate from the posterior aspect of the IVC and measured approximately 8 cm in length by 4 cm in width, with the widest part lying within the right atrium. Ten minutes of circulatory arrest was required and the tumor was excised along with a portion of the posterior inferior vena cava. The tumor mass was well demarcated with gross total resection. Of note, tumor margins at the time of resection were negative. The inferior vena cava was then closed primarily from the intraluminal side. Surgical clips were left in place to help identify the target for radiation therapy. In addition, the patient had her PFO surgically corrected. The patient tolerated the procedure well and was weaned from bypass without difficulty.

Postoperatively, the patient received radiation therapy. The patient received a course targeting the resection bed of the origin of her tumor in the inferior vena cava. She was treated with photons of 18 megavolts (MV) at the isocenter at 2 Gray (Gy) per fraction for 25 fractions to a total dose of 50 Gy. This was followed by an additional boost to a small area for an additional five fractions to a total dose 10 Gy. The total dose to the tumor bed was 60 Gy. The patient tolerated the course of radiation well and reported no side effects, such as skin toxicity or gastrointestinal toxicity. There were no breaks during treatment.

Subsequent clinical examinations every 6 months and imaging with yearly CT scans have confirmed that the patient has remained disease free 5 years after treatment.

## Discussion

Tumors such as renal carcinomas, pheochromocytomas, hematomas or testicular tumors can secondarily invade the vena cava. Leiomyosarcomas of the inferior vena cava are primary vascular tumors that are relatively rare [1]. They are most frequently seen in the sixth decade with a female predominance [2]. Metastatic disease can involve the liver, lung, lymph nodes, or bone. However, metastases have been reported in fewer than 50% of cases [4].

Clinical findings are nonspecific and may precede the diagnosis by several years [3]. Initial symptoms are often relatively minor. The three most common presenting symptoms are abdominal pain (52%), distention (20%), and deep venous thrombosis (12%) [5]. Other findings include the Budd-Chiari syndrome, a new heart murmur, signs of cerebral embolism, and pain in the abdomen and/or the back.

In 1992 the International Registry of Inferior Vena Cava Leiomyosarcomas collected 218 cases [1]. In that series, tumors arose from the IVC lower segment in 80 patients, from the middle in 94, and from the upper IVC in 41 [1]. IVC leiomyosarcomas arising below the renal vein (segment I) cause pain in the right-lower quadrant, back, and flank as well as causing varying degrees of lower extremity edema [4]. Those arising in the middle caval segment (segment II) cause right-upper-quadrant pain and sometimes renovascular hypertension [4]. Those arising above the hepatic vein (segment III) cause varying manifestations of the Budd-Chiari syndrome [4].

Modern imaging modalities using computed tomography, magnetic resonance imaging, individually or in combination with cavography, ultrasound, and echocardiography, allow for an early and accurate preoperative diagnosis, resulting in a higher rate of surgical resection and improved survival [2,3,5]. It is also speculated that the recent progress of diagnostic investigations, such as spiral CT with reconstruction and, positron emission tomography (PET), will be able to even further define tumor extent and aid in defining the most appropriate treatment approach [6]. In the present case, the PET scan showed mild abnormal glucose consumption compatible with a malignancy such as sarcoma and represents the first reported case of leiomyosarcoma of the IVC imaged by PET/CT.

Despite advancing imaging technology, a biopsy is still indicated for formal diagnosis. Histopathology of leiomyosarcoma reveals spindle tumor cells, which are positive for markers of smooth muscle activity including vimentin, muscle actin, alpha-smooth muscle actin, and desmin [7].

Because of limited experience with this disease, the optimal management of IVC leiomyosarcoma is unknown [8]. Aggressive surgical treatment is recommended in light of the tumor's slow growth pattern and relatively low metastatic potential [4]. There is limited experience in the literature regarding surgical management of these IVC tumors and limited data on the long-term survival of these patients. Complete surgical resection with a tumor-free margin (1 cm) is felt to be the treatment of choice [2]. A case series of 25 patients with primary IVC leiomyosarcoma treated between 1982 and 2002 showed that complete resection of primary IVC leiomyosarcomas was feasible and that the IVC could be managed either by primary repair or ligation with a low risk of severe postoperative edema [5]. Resection of the IVC with prosthetic reconstruction allows for complete tumor resection and provides durable relief from symptoms of venous obstruction [8]. Tumors involving the lower IVC are most amenable to surgery while upper caval leiomyosarcomas are the least amenable to complete removal. Extensive collateral venous drainage of the left kidney preserves renal function during resection of middle caval tumors [4].

Neoadjuvant therapy may be given to downsize the tumor and increase resectability rates. Nonetheless, when complete resection is not possible, debulking combined with radiation therapy still provides good palliation [2]. In another series, 14 patients with leiomyosarcoma of the IVC were treated wide resection between 1978 and 1997; in this series, radiation therapy diminished local recurrence and improved median survival (6 months in 2 patients without irradiation compared to 51 months in 12 irradiated patients) [9]. Patients who received combined chemotherapy and radiation lived longer than those who received radiation therapy alone (P < 0.05) [9]. Chemotherapy, using adriamycin-ifosfamide or chemotherapy combined with radiation therapy offer potential adjuvant treatment options [10]. The 5-year cumulative survival rate was 53% for patients with leiomyosarcoma of the IVC suggesting that aggressive surgical management combined with adjuvant therapy offers the best treatment for patients with leiomyosarcoma of the IVC [9].

The International Registry of Inferior Vena Cava (IVC) Leiomyosarcomas analyzed their cohort of patients for predictive factors of clinical outcome. A radical tumor resection was undertaken in 134 (61.5%) patients, 26 (11.9%) had a palliative resection, and 58 (26.6%) were inoperable [1]. An increased risk of death was associated with upper IVC segment involvement (p < 0.001), lower limb edema (p < 0.001), Budd-Chiari syndrome (p < 0.001), intraluminal tumor growth (p < 0.001) and IVC occlusion (p < 0.001) [1]. Radical tumor resection was associated with better 5- and 10-year survival rates (49.4% and 29.5%) when compared to patients undergoing palliative resection or those who were inoperable [1]. Data published in 2003 showed that patients undergoing complete resection had 3- and 5-year survival rates of 76% and 33% respectively [5]. Tumors that arose from the middle segment fared better than those of the lower segment (p < 0.002) in one case series [1]. The absence of a palpable abdominal mass (p < 0.03) or abdominal pain (p < 0.04) was also associated with a better outcome and longer survival [1]. Another case review of 14 patients found that age, gender, tumor size, tumor grade, and lymph node status did not impact survival of patients with leiomyosarcoma of the IVC [9].

In these patients, overall prognosis remains poor with a mean survival of 2 years but ranging from a few weeks to eight years [4]. Even in this population, the risks and benefits of palliative resection must still be considered as significant symptomatic relief has been reported.

## Conclusion

Leiomyosarcomas are the most common malignancy involving the IVC. Although correlations between clinical manifestations and the location of the tumor within the IVC have been noted, patients often present with non-specific symptoms such as dyspnea, malaise, weight loss, and abdominal or back pain. Fluoroscopically guided biopsy is required for diagnosis, but various imaging modalities have allowed for earlier diagnosis and, subsequently, better outcomes. A combined PET/CT scan can be used for staging and to assist with treatment planning. Though chemotherapy and/or radiation therapy may serve as adjuncts to surgical resection, aggressive surgical treatment is currently recommended due to the tumor's slow growth pattern and low metastatic potential.

## Abbreviations

IVC: inferior vena cava; PFO: patent foramen ovale; PET: positron emission tomography; TTE: transthoracic echocardiogram; US: ultrasound.

## Consent

Written informed consent was obtained from the patient for publication of this case report and accompanying images. A copy of the written consent is available for review by the Editor-in-Chief of this journal.

## Competing interests

The authors declare that they have no competing interests.

## Authors' contributions

VPR, PJV, RWD, and DGM drafted the manuscript and prepared the figures. GFM, JPB, SKW, and LK reviewed and amended the manuscript. All authors read and approved the final manuscript.

## References

[B1] Mingoli A Cavallaro A Sapienza P Di Marzo L Feldhaus RJ Cavallari N International registry of inferior vena cava leiomyosarcoma: analysis of a world series on 218 patients Anticancer Res 1996 16 5B 3201 3205 8920790

[B2] Hemant D Krantikumar R Amita J Chawla A Ranjeet N Primary leiomyosarcoma of inferior vena cava, a rare entity: Imaging features Australas Radiol 2001 45 4 448 451 10.1046/j.1440-1673.2001.00955.x 11903177

[B3] Gowda RM Gowda MR Mehta NJ Osborne R Bixon R Vasavada BC Sacchi TJ Right atrial extension of primary venous leiomyosarcoma: pulmonary embolism and Budd-Chiari syndrome at presentation-a case report Angiology 2004 55 2 213 216 10.1177/000331970405500215 15026878

[B4] Griffin AS Sterchi JM Primary leiomyosarcoma of the inferior vena cava: a case report and review of the literature J Surg Oncol 1987 34 1 53 60 10.1002/jso.2930340114 3807376

[B5] Hollenbeck ST Grobmeyer SR Kent KC Brennan MF Surgical treatment and outcomes of patients with primary inferior vena cava leiomyosarcoma J Am Coll Surg 2003 197 4 575 579 10.1016/S1072-7515(03)00433-2 14522326

[B6] Babatasi G Massetti M Agostini D Galateau F Le Page O Saloux E Bhoyroo S Grollier G Potier JC Khayat A Leiomyosarcoma of the heart and great vessels Ann Cardiol Angeiol 1998 47 7 451 458 9772966

[B7] Nikaido T Endo Y Nimura S Ishikura H Ushigome S Dumbbell-shaped leiomyosarcoma of the inferior vena cava with foci of rhabdoid changes and osteoclast-type giant cells Pathol Int 2004 54 4 256 260 10.1111/j.1440-1827.2004.01616.x 15028027

[B8] Sarkar R Eilber FR Gelabert HA Quinones-Baldrich WJ Prosthetic replacement of the inferior vena cava for malignancy J Vasc Surg 1998 28 1 75 81 10.1016/S0741-5214(98)70202-2 9685133

[B9] Hines OJ Nelson S Quinones-Baldrich WJ Eilber FR Leiomyosarcoma of the inferior vena cava: prognosis and comparison with leiomyosarcoma of other anatomic sites Cancer 1999 85 5 1077 1083 10.1002/(SICI)1097-0142(19990301)85:5<1077::AID-CNCR10>3.0.CO;2-0 10091791

[B10] Kieffer E Alaoui M Piette JC Cacoub P Chiche L Leiomyosarcoma of the inferior vena cava: experience in 22 cases Ann Surg 2006 244 2 289 295 10.1097/01.sla.0000229964.71743.db 16858193 PMC1602179

